# Supplementary material for: Maternal and Fetal Bile Acid Homeostasis Regulated by Sulfated Progesterone Metabolites through FXR Signaling Pathway in a Pregnant Sow Model
Source: Int J Mol Sci. 2022 Jun 10;23(12):6496. doi: 10.3390/ijms23126496 (PMC9224516; doi:10.3390/ijms23126496)
Supplement: Supplementary file 1 [file ijms-23-06496-s001.zip › Figure S1-S2.pdf]

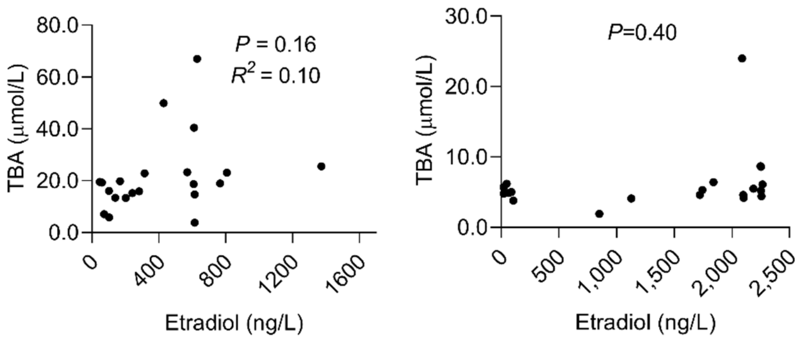

(a) (b)

Figure S1. (a) The correlation of maternal estradiol and its serum TBA at G60, G90 and L0 (n=21), (b) the correlation of fetal estradiol and its serum TBA at G60, G90 and L0 (n=5-6/group).

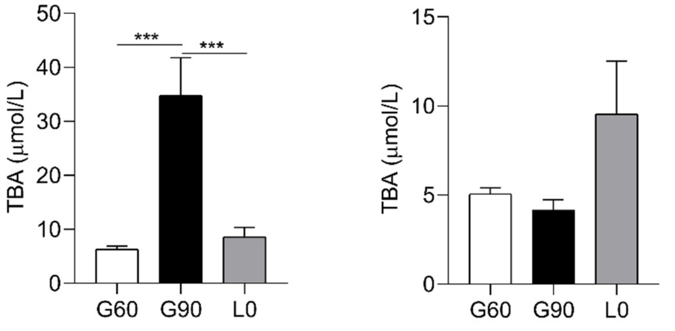

(a) (b)

Figure S2. (a) The maternal serum TBA levels at G60, G90 and L0, (b) the fetal serum TBA levels at G60, G90 and L0. n=5-6/group.

Data are shown as means ± SE, **P* <0.05, ***P* <0.01, ****P*<0.001.
