# Supplementary material for: Maternal and Fetal Bile Acid Homeostasis Regulated by Sulfated Progesterone Metabolites through FXR Signaling Pathway in a Pregnant Sow Model
Source: Int J Mol Sci. 2022 Jun 10;23(12):6496. doi: 10.3390/ijms23126496 (PMC9224516; doi:10.3390/ijms23126496)
Supplement: Supplementary file 1 [file ijms-23-06496-s001.zip › Table S1.pdf]

**Table S1. Primer sets for real-time RT-PCR analysis**

| Genes | Primer sequence（5'-3'） | Accession number |
|---|---|---|
| *β-actin* | FW AGAGCAAGAGAGGCATCCTG | XM_003124280.5 |
| | RV CACGCAGCTCGTTGTAGAAG | |
| *CYP7A1* | FW GAAAGAGAGACCACATCTCGG | NM_001005352 |
| | RV GAATGGTGTTGGCTTGCGAT | |
| *CYP8B1* | FW CCGGAAGAATATGTTGGAAT | NM_214426.1 |
| | RV AAGTCTAGTTTTCTCTTCGC | |
| *NTCP/SLC10A1* | FW ACTTTCGGAAACCTAAGGGACT | XM_001927695.5 |
| | RV AAGAGCTTGCCCAGTGCAAAG | |
| *OSTβ/SLC51B* | FW GAAATCCAAAGACGCTGCCA | XM_005658570.3 |
| | RV CCCTTAGGATGGTCAGGTTGT | |
| *BSEP/ABCB11* | FW TTTCATTCAGCGCCTGACCA | XM_003133457.5 |
| | RV ACTCCAATGAGAGGGCTGAC | |
| *MRP2/ABCC2* | FW TGCAAGTACGGACCAGTGTC | XM_021073710.1 |
| | RV AACGGTGTACTGCTTCCTGG | |
| *MDR3/ABCB4* | FW CCAGGAAGCAAAGAAACTCAATG | XM_013989596.2 |
| | RV CTCCTCCAGGGTCACAATGC | |
| *SULT2A1* | FW CCATGCGAGACAAGGAGAAC | NM_001037150.1 |
| | RV CATGACCTGGAAGGAGCTGT | |
| *FXR* | FW ATACAACAGTGTTCCGTTTC | NM_001287412.1 |
| | RV AGAGTCTCAGCAGGCATT | |
| *SHP* | FW GCCTACCTGAAAGGGACCAT | DQ002896 |
| | RV CAACGGGTGTCAAGCCTTTA | |
| *FGF19* | FW AGTACTCGGATGAGGACTGTGCTT | XM_003122420.3 |
| | RV AGAGACGGGCAGATGGTGTTTCTT | |
